# Optimized statistical parametric mapping procedure for NIRS data contaminated by motion artifacts: Neurometric analysis of body schema extension

**DOI:** 10.1007/s40708-017-0070-x

**Published:** 2017-07-29

**Authors:** Satoshi Suzuki

**Affiliations:** Department of Robotics and Mechatronics, Tokyo Denki University, 5 Asahi-Chou, Senju, Adachi-ku, Tokyo, 120-8551 Japan

**Keywords:** Body schema, Near-infrared spectroscopy, General linear model, Statistical parametric mapping, Hand-tracing task, Motion artifacts

## Abstract

This study investigated the spatial distribution of brain activity on body schema (BS) modification induced by natural body motion using two versions of a hand-tracing task. In Task 1, participants traced Japanese Hiragana characters using the right forefinger, requiring no BS expansion. In Task 2, participants performed the tracing task with a long stick, requiring BS expansion. Spatial distribution was analyzed using general linear model (GLM)-based statistical parametric mapping of near-infrared spectroscopy data contaminated with motion artifacts caused by the hand-tracing task. Three methods were utilized in series to counter the artifacts, and optimal conditions and modifications were investigated: a model-free method (Step 1), a convolution matrix method (Step 2), and a boxcar-function-based Gaussian convolution method (Step 3). The results revealed four methodological findings: (1) Deoxyhemoglobin was suitable for the GLM because both Akaike information criterion and the variance against the averaged hemodynamic response function were smaller than for other signals, (2) a high-pass filter with a cutoff frequency of .014 Hz was effective, (3) the hemodynamic response function computed from a Gaussian kernel function and its first- and second-derivative terms should be included in the GLM model, and (4) correction of non-autocorrelation and use of effective degrees of freedom were critical. Investigating *z*-maps computed according to these guidelines revealed that contiguous areas of BA7–BA40–BA21 in the right hemisphere became significantly activated ($t(15); p<.001$, $p<.01$, and $p<.001$, respectively) during BS modification while performing the hand-tracing task.

## Introduction

In the human brain, peri-personal space [1] is represented by embedding the spatial volume of external objects, such as a hat (clothing) or a stick (tool), into an internal body map [2]. In this process, people typically feel the object as an extension of their own body [3]. The mechanism underlying this sophisticated cognitive process is known as body schema (BS) modification [4]. This mechanism is considered a form of homuncular flexibility, involving constant changes to the shape of the homunculus, which is an approximate internal map of the human body in the cortex that is often visualized as a distorted human body [5]. The concept of the BS was initially proposed by Head and Holmes [6], who defined it as a postural model of the body that actively organizes and modifies the impressions produced by incoming sensory impulses [6]. The existence of the BS was confirmed in the 1990s by analyzing brain function in macaque monkeys [7]. The BS is considered to be vital for spatial cognitive function and is associated with various brain areas, including the sensorimotor cortex [8], Broca’s area (BA44), the inferior parietal lobule (BA40) [9], the primary motor cortex (BA4) [10], and the mirror neuron system [11]. One experimental approach to examining the BS involves the induction of a “confused” brain state by presenting mismatching visual and haptic stimuli, as in the rubber hand illusion (RHI) [12, 13]. Similar variations, such as the visual–proprioceptive synchrony judgment task [14] and the visual–proprioceptive mismatch task [15], have also been examined. Other studies utilized motion illusions to examine the BS more directly. Motion illusions arise when somatic sensations are confused by physically vibrating the muscle spindle that provides axial and limb position information to the central nervous system [16]. Examples include illusory arm movement [17], the Pinocchio illusion [18], and the waist-shrinking illusion [19]. Electrical stimuli applied to the skin can also induce similar motion illusions [20].

In addition, the BS plays a significant role in the ability to drive a vehicle [21]. For example, a person’s sense of car width is a form of BS modification [22]. For a skilled driver, the whole spatial volume of the vehicle body is perceived as an extension of the driver’s peri-personal space [23]. Such spatial cognitive function is also involved in teleoperation systems that require the operator to manipulate a machine remotely [24]. In both driving a car and remote operation of a robot, the machine (car or robot) must be manipulated like one’s own body. This sensation of body ownership is a type of BS modification [25, 26].

Additionally, the BS is heavily involved in some cognitive disorders [27]. Alice in Wonderland syndrome (involving distorted awareness of body size, mass, or its position in space), autotopagnosia (involving mislocalization of body parts and bodily sensations), and phantom sensation (awareness of an amputated limb) are examples of such disorders. Because the BS is related to such varied human functions, a quantitative method for evaluating the strength of BS modification may be useful both for rehabilitation of spatial cognition disorders and for the estimation of spatial cognitive skill during vehicle operation. Possible BS measurement methods such as functional magnetic resonance imaging (fMRI), positron emission tomography (PET), and magnetoencephalography (MEG) are, however, not adequate for evaluation, because they all require the participant’s head to be fixed to a stationary measurement unit mounted on the floor. Natural BS modification is difficult to induce using such stationary measurement devices because participants cannot move their bodies freely. Near-infrared spectroscopy (NIRS) is an alternative measurement method in which the measurement unit can be attached to the participant’s head while still permitting head movement. NIRS may thus have useful applications in daily life, and portable NIRS systems have recently been marketed commercially.

While NIRS may be an appropriate measurement technique for measuring brain activity during daily tasks, the relationship between NIRS activity and modifications to the BS is not currently understood. While a brain map of BS modification would be useful, no analysis procedure for constructing a map from NIRS data contaminated by motion artifacts has been established to date. Even mild motion, such as an arm movement, causes strong artifacts in NIRS data. As such, there are several experimental limitations involved in current NIRS methods: the need for participants to maintain a sitting posture, the restriction of movement to the right upper arm only, the inability to twist one’s head, and the need to avoid conversation, all of which may induce cognition-related brain activity that contaminates NIRS data.

Statistical parametric mapping (SPM) has recently become a popular method for investigating the spatial distribution of brain activity [28] in studies using fMRI and PET. Several studies describing the application of SPM to NIRS data have been reported [14, 29–31]. According to the SPM procedure, characteristics of brain activity are identified statistically using a general linear model (GLM) [32] to evaluate the accuracy of fit of brain activity against a canonical response pattern of cerebral blood flow. Random effects are then analyzed using the accuracy of fit. Because of the effort expended in recent decades to develop the SPM software package [33], this approach has been established as a standard analysis method for fMRI and PET interpretation. SPM is thus becoming the de facto standard for examining common features in human brain activity. It is widely used for investigating brain functions that relate to a wide brain area.

However, unlike fMRI and PET, various adjustments of experimental design analysis are required in NIRS studies, because of the following issues:

*Issue 1*: To be analyzed with the GLM, signals must satisfy the assumption of normal distribution [32]; however, the actual responses of regional cerebral blood flow (rCBF) are not necessarily normally distributed.
*Issue 2*: It is difficult to satisfy the GLM assumption of non-autocorrelation of errors, since rCBF is time dependent [14].
*Issue 3*: It is challenging to distinguish meaningful low-frequency components in rCBF from true noise, such as drift and bias.

These issues have often been implicitly ignored in previous studies because the default parameters of the SPM software were applied without careful consideration [31].

In addition, motion artifacts strongly affect NIRS data when analyzing brain activity that accompanies body motion. As such, consideration of body motion is inevitable because natural body motion is required to combine the visual and haptic senses that are involved in BS. Importantly, ill-conditioned data arising from motion artifact contamination cannot satisfy the requirements of the GLM–SPM because well-conditioned data are implicitly required for comparison with the canonical waveform. For this reason, most previous studies of NIRS–SPM have utilized experimental paradigms that prohibit body motion (as in fMRI and MEG studies). As such, these methods cannot be directly used to analyze BS modification accompanying motion artifacts. Overall, the brain areas associated with BS modification induced by spontaneous body motion have not yet been comprehensively examined in humans, although these mechanisms have been identified in the monkey brain [7], and fragmentary human evidence has been reported [9, 11].

Consequently, existing studies of NIRS–SPM have been unable to use the unique benefit of the NIRS method, which permits movement of the head and body. Therefore, the current study sought to establish a practical NIRS–SPM procedure using a task that requires the control of hand and arm movements. The main aims of this study were as follows:

1. Creating guidelines for using NIRS–SPM to analyze rCBF accompanied by body motion artifacts.
2. Quantifying the spatial distribution of human brain activity during BS modification induced by natural and spontaneous body motion.

Concerning the first aim, several conditions and modifications were investigated using the following three steps: Step (1) a model-free method analyzing cerebral blood volume (CBV), Step (2) a convolution matrix method known as the orthodox GLM, and Step (3) a boxcar-function-based Gaussian convolution method.

## Experiment

### Hand-tracing task

**Fig. 1:**
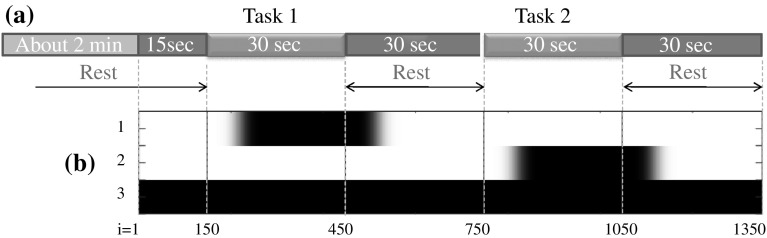
**a** Experimental time sequence, **b** design matrix ${\mathbf {X}}$ for the GLM: **b** shows the elements in the design matrix ${\mathbf {X}}$ in *black* (${\hbox {value}}=1$) and *white* (${\hbox {value}}=0$) in the *gray image*. Refer to Sect. 3.2 for details of the design matrix

A hand-tracing task was devised to examine differences in brain activity related to BS modification. In this task, participants were instructed to trace the curve of Japanese Hiragana characters that were printed on paper (Task 1) or projected onto a screen (Task 2). In Task 1, participants used the right forefinger to trace the characters. In Task 2, participants used a 1.5-m stick held in the right hand to trace characters that were projected 2.0 m in front of them. Importantly, Task 1 entails the use of the BS of participants’ own body only, whereas Task 2 involves an extension of the BS to the tip of the long stick. During both tasks, participants sat on a chair, and the sitting position was adjusted so that the participant could touch the characters with the tip of the finger or stick. The size of projected characters was enlarged in proportion to the distance to the screen to keep the perturbation of hand motion similar in both tasks. To avoid inducing unnecessary brain activation from environmental light and sound, participants performed the tasks while wearing noise-canceling headphones inside a tent covered with a curtain. Thirty-second rests were given after each task, as shown in Fig. 1a. The investigator touched the shoulder of the participant to signal the start and end of each task.

### NIRS measurement

**Fig. 2:**
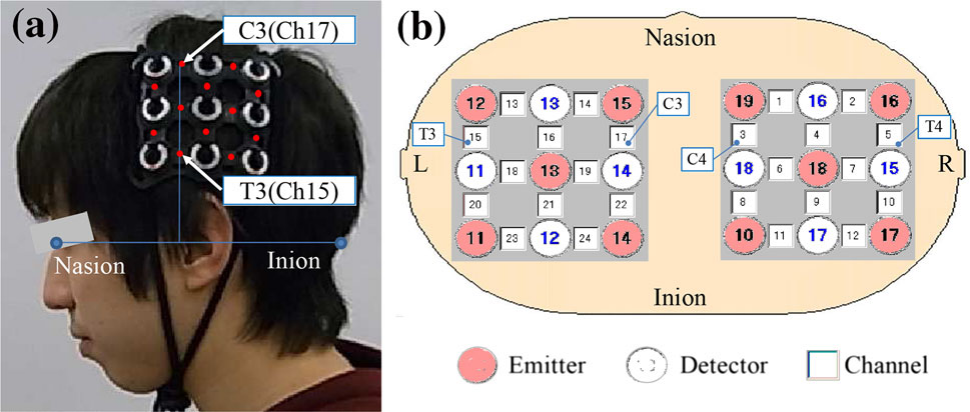
**a** Locations of probes, **b** channel layout

Previous evidence suggests that performing a hand-tracing task would be likely to involve activation in the primary motor area (M1), the primary somatosensory area (S1), and the premotor area (PM) [34–37], because the action involved in hand tracing requires the control of hand and arm movements. The activation of these brain areas alone, however, does not provide sufficient evidence to identify BS modification. Hence, the present study also examined the inferior parietal lobule (BA40). This brain region is not thought to directly relate to hand motion, but is one of the cortical areas associated with BS [9]. Therefore, in the current experiment, areas around BA40 were monitored using ETG-4000 (Hitachi Medico, Tokyo, Japan) using two $3 \times 3$ holders, as shown in Fig. 2. A total of 24 data channels were measured. The NIRS probes were attached to participants’ heads using the international 10–20 system so that C3(4) was located at the forefront of the upper array of the holder, and the second vertical line of the holder was perpendicular to the nasion–inion line, as shown in Fig. 2a. Thus, C3(C4) and T3(T4) on the left (right) hemispheres corresponded to Ch.17(Ch.3) and Ch.15(Ch.5), respectively.

The content and procedure of the experiment were approved by the Tokyo Denki University Human Bioethics Review Committee, and experiments were conducted after explaining the experiment to the participants in full and receiving written consent. Sixteen healthy university students (20–23 years old) participated ($N=16$) in this experiment.

## Analyses

To determine the optimal conditions for NIRS–SPM analysis dealing with rCBF contaminated by motion artifacts, Steps 1–3 were applied to the rCBF data, in sequence. Step 1 examined the rCBF waveform to determine a hemodynamic response function (HRF) candidate and tentatively select the NIRS hemoglobin type for the GLM analysis. In Step 2, a low-frequency noise that causes adverse effects on fitting time-sequential rCBF data to the GLM was eliminated, and the degrees of freedom of the SPM computation were modified in order to obtain correct statistical results. In Step 3, an adequate canonical model in GLM was found, to enhance the accuracy of fit, and an optimal condition for the NIRS–SPM was derived after autocorrelation modification and the final choice of hemoglobin type were identified. These steps fundamentally adhere to the following basic stages of a general SPM approach [28]:

*First-level analysis*: A statistical test ascertains whether the rCBF shows significantly different responses according to the task condition with respect to each measurement channel (one-sample *t* test).
*Second-level analysis*: After statistics obtained in the first-level analysis are converted into *z*-values, an average of the population to which the *z*-values of all participants belong is tested (random-effects analysis) [38].

The details of these steps and the analysis results are explained below, in sequence.

### Step 1: Model-free method

The increase of total hemoglobin (Hb) has often been used to investigate brain activity in previous studies. However, other wave patterns such as “both oxy-Hb and total-Hb decrease” and “oxy-Hb, deoxy-Hb, and total-Hb increase” have also been used [39]. In the case of young adults, total-Hb concentration tends to reflect both oxy and deoxy changes, since the increase in oxy-Hb tends to be larger than the decrease in deoxy-Hb [39].

It is, however, difficult to select a consistent pattern because these Hb-based parameters also differ according to the age of the participant, the purpose of the research, measurement conditions, and the researchers’ preferences. In practice, oxy-Hb is considered by some researchers to be adequate for detecting local brain activity [14, 40, 41], while other studies [29, 42, 43] support the use of deoxy-Hb. Therefore, the first step in this study was to investigate the most appropriate type of hemoglobin using a model-free approach unrelated to the HRF.

Close examination of rCBF responses revealed that regional cerebral blood volume (rCBV) in several channels decreased at the beginning of Task 1 and increased for several seconds at the beginning of Task 2. Based on this observation, a null hypothesis of no difference was tested using a paired *t* test against two values of rCBV, referred to as $S_{1 \cdot \tau }$ and $S_{2 \cdot \tau }$. These values were computed by integrating the rCBF data for $\tau$ seconds from the beginning of each task.

1 $$\begin{aligned} S_{l \cdot \tau }= \sum _{i=0}^{\tau /\Delta } (y_l(i)-b_l) / (\tau /\Delta ) \ \ (l=1,2) \end{aligned}$$

2 $$\begin{aligned} b_l&:= \sum _{i=-\lfloor 5/\Delta \rfloor }^{0} y_l(i) / \left( \lfloor 5/\Delta \rfloor \right) , \end{aligned}$$

where $y_l(i)$ is the rCBF data at the sampling count *i* on Task *l* from the beginning of the task, the sampling interval $\Delta$ is .1 s, $b_l$ is a bias computed by averaging 5 s of data just before the beginning of task, and an operator $\lfloor * \rfloor$ is a floor function. The *z*-values converted from statistics computed in this paired *t* test are shown in Table 1. The *z*-values were computed for each channel using all participants’ data ($N=16$), and other results obtained using different $\tau$ values are summarized in the same table. This table shows an existence of significant differences in channels 4–10, 12, and 15 for all types of Hb. This result demonstrates that Tasks 1 and 2 induced significantly different brain activity responses.

**Table 1:**
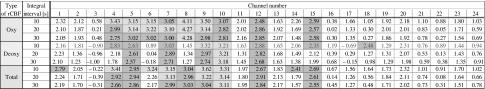
Results of statistical tests using a model-free method in Step 1: *z*-values obtained using a paired *t* test, $df=15$

Blue cells show $z>2.33\, (p<.01)$, and red cells show $z>3.09\, (p<.001)$. (Color table online)

**Fig. 3:**
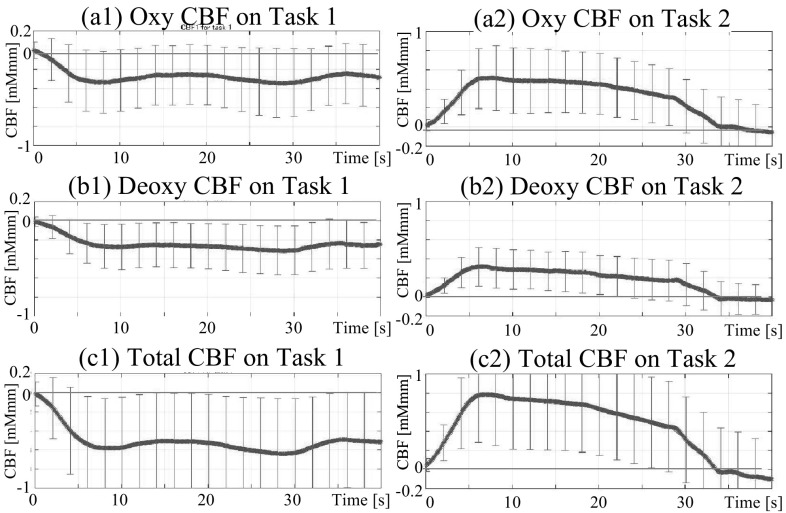
Averaged waveform of hemodynamic responses at channels showing significant differences in Task 1 (*left graphs*) and Task 2 (*right graphs*). *Error bars* in each graph indicate $.5 \sigma$ of the measured waveforms every 10 s

The current method was a relatively simple process, and the results shown in Table 1 may possess lower reliability because the parameters [an integral interval $\tau$ in Eq. (1) and a duration to compute the bias $b_l$ in Eq. (2)] were determined subjectively without a theoretical guarantee of optimality, despite consideration of the task condition and the actual NIRS response data. This method, however, contributes to an improved understanding of a tendency in a wide area of common brain activity from multi-channel measurement data. Thus, we assumed the results shown in Table 1 were provisional, and used them to judge the validity of the assumption of the existence of a certain HRF wave pattern for the subsequent Steps 2 and 3, which were processed from objective criteria. Because the data in this table indicate that the channels responding to the difference between Tasks 1 and 2 were ch. 4–10, 12, and 15, a canonical waveform of rCBF was computed using the rCBF data measured in these channels for the HRF. That is, we averaged all rCBF data measured in these channels during each task period into one waveform for each task. The results of the canonical forms are shown in Fig. 3. All graphs in the figure show rounded trapezoidal waveforms similar to the convolved response of a boxcar and a Gaussian function. The results revealed that rCBF during Task 1 decreased [see graphs (a1) (b1)] while rCBF during Task 2 increased [see graph (a2) (b2)] for both oxy- and deoxy-Hb. These findings indicated that a waveform similar to that used in previous studies, involving the convolved response of a boxcar and a Gaussian function, was appropriate for the current analysis. In addition, the averaged rCBF waveform in all cases demonstrated substantial variance because of motion artifacts, as indicated by the long error bars in each graph. Notably, the variance in deoxy-Hb was half that of oxy-Hb [as indicated by the difference in the length of the error bars between graph (a1) and graph (b1), and between graph (a2) and graph (b2)]. This result indicates that deoxy-Hb was less affected by individual differences than oxy-Hb.

Taken together, these findings indicate that deoxy-Hb may be better suited for GLM analysis using the convolved response of a boxcar and a Gaussian function for analysis of rCBF affected by motion artifacts. Therefore, the deoxy-Hb NIRS data were mainly analyzed provisionally in the following analyses. The validity of the selection of deoxy-Hb was judged in Steps 2 and 3.

### Step 2: Convolution matrix method

After Step 1, a GLM–SPM method presented in [44] called the convolution matrix method was applied to the deoxy-Hb responses because the validity of assuming a HRF was confirmed in Step 1, as the procedure presented in [44] is considered a basic version of various extended GLM–SPM methods. To examine differences between the two versions of the hand-tracing task, the following GLM equation was assumed using independent variables $x_k\ (k=1,2)$ and a response variable *y*.

3 $$\begin{aligned} {}^{j}y(i) = {}^{j}b_1\cdot x_1(i)+{}^{j}b_2\cdot x_2(i) +{}^{j}d+{}^{j}e(i), \end{aligned}$$

where $j\ (=1,\ldots ,24)$ is an index of channel, $b_k$ are unknown coefficient parameters (const.), *d* is a drift term (const.), and *e* is a residual that is assumed to be independently and identically distributed normally with a mean of zero. The maximum of the sampling counter *i* and the total number of independent variables $x_k$ are denoted as *I* and *K*, respectively. Equations defined by Eq. (3) for all *i* are summarized in the following matrix form:

4 $$\begin{aligned} {}^{j}{\mathbf {Y}}= {\mathbf {X}}\cdot {}^{j} {\mathbf {B}}+ {}^{j}{\mathbf {E}}, \end{aligned}$$

where ${\mathbf {X}}\in {{\mathbb {R}}}^{I \times (K+1)}$ is a design matrix, ${\mathbf {Y}}\in {{\mathbb {R}}}^{I}$ is a response vector, ${\mathbf {B}}\in {\mathbb {R}}^{(K+1)}$ is a parameter vector, ${\mathbf {E}}\in {\mathbb {R}}^{I}$ is a residual vector, and ${\mathbf {X}}$ and ${\mathbf {B}}$ are defined as

5 $$\begin{aligned} {\mathbf {X}} := \left[ \begin{array}{ccc} x_1(1) &{} \quad x_2(1) &{} \quad 1 \\ \vdots &{}\quad \vdots &{}\quad \vdots \\ x_1(I) &{} \quad x_2(I) &{} \quad 1 \\ \end{array} \right] , \quad {}^j {\mathbf {B}} := \left[ \begin{array}{c} {}^j b_1 \\ {}^j b_2 \\ {}^j d \end{array} \right] . \end{aligned}$$

*First-level analysis* This step investigated whether the hemodynamic responses differed depending on the task conditions by statistically investigating the magnitude of estimations of coefficients in Eq. (3) with respect to each channel for each participant. The details of this technique are explained below.

First, the design matrix ${\mathbf {X}}$ was defined using a time-series signal of a boxcar function with a value of 1 during the task period and a value of 0 otherwise. Second, a convolution matrix ${\mathbf {H}}\in {\mathbb {R}}^{(I+M) \times I}$ was defined using a Gaussian function to approximate the response of the rCBF. Estimates $\hat{{\mathbf {B}}}$ for ${\mathbf {B}}$ were computed as follows, using the ordinary least squares (OLS) method [44].

6 $$\begin{aligned} {}^{j}\hat{{\mathbf {B}}}&= \left( {\mathbf {X}}_{a}^{{\mathrm{T}}} {\mathbf {X}}_{a}\right) ^{-1} {\mathbf {X}}_{a}^{{\mathrm{T}}} {\mathbf {H}}\cdot {}^{j} {\mathbf {Y}}\nonumber \\{\mathbf {X}}_{a}&:= {\mathbf {H}}{\mathbf {X}}\end{aligned}$$

Specifically, the convolution matrix ${\mathbf {H}}$ was designed using a Gaussian function with a 4.0-s full width half maximum $({ FWHM}=4.0)$ [29], and the total length of ${\mathbf {Y}}$ was specified as 135 s (i.e., $I=1350$) because the data range including the 15- and 30-s rests at the beginning of Task 1 and the end of Task 2, respectively, was examined, as shown in previous Fig. 1b. Finally, for a statistical test of the estimates $\hat{{\mathbf {B}}}$, a contrast matrix was chosen as ${\mathbf {C}}=[\ -1\ 1\ 0\ ]$, and the Wald statistic ($={\mathbf {C}}\hat{{\mathbf {B}}}$/standard error of slope coefficient) [45] was computed for each channel and tested with a one-sample *t* test. Importantly, the usual degrees of freedom (DoF) used in common GLM methods computed by $(I-{\mathrm {rank}}({\mathbf {X}}))$ [46] are likely to overestimate the statistic [44] because large statistical values are computed inaccurately when long time-series data are analyzed. To avoid this issue, we used the following alternative Wald statistic *t*, which was modified using an effective DoF [47]

7 $$\begin{aligned} t = \frac{{\mathbf {C}}\cdot {}^j \hat{{\mathbf {B}}}}{\sqrt{\epsilon ^2 {\mathbf {C}}\left( {\mathbf {X}}_{a}^{{\mathrm{T}}} {\mathbf {X}}_{a}\right) ^{-1} {\mathbf {X}}_{a}^{{\mathrm{T}}} {\mathbf {V}}{\mathbf {X}}_{a}\left( {\mathbf {X}}_{a}^{{\mathrm{T}}} {\mathbf {X}}_{a}\right) ^{-1} {\mathbf {C}}^{{\mathrm{T}}}},} \end{aligned}$$

where ${\mathbf {V}}:={\mathbf {H}}{\mathbf {H}}^{{\mathrm{T}}}$ and $\epsilon ^2 \in {\mathbb {R}}^1$ is an unbiased estimator. Here $\epsilon ^2$ is computed from a residual-forming matrix ${\mathbf {R}}\in {\mathbb {R}}^{(I+M)\times (I+M)}$ and a vector of residuals ${\mathbf {r}}\in {\mathbb {R}}^{(I+M)}$ as

8 $$\begin{aligned} \epsilon ^2&= {\mathbf {r}}^{{\mathrm{T}}} {\mathbf {r}}/ {\mathrm {trace}}({\mathbf {R}}{\mathbf {V}})\nonumber \\{\mathbf {r}}&:= {\mathbf {R}}{\mathbf {H}}\cdot {}^j {\mathbf {Y}}\nonumber \\{\mathbf {R}}&:= {\mathbf {I}}- {\mathbf {X}}_{a}\left( {\mathbf {X}}_{a}^{{\mathrm{T}}} {\mathbf {X}}_{a}\right) ^{-1} {\mathbf {X}}_{a}^{{\mathrm{T}}} . \end{aligned}$$

Although Eq. (7) can be computed without the direct use of the effective DoF *v*, the value of *v* was required to convert the *t* into a *z*-value during the second-level analysis. Hence, *v* was computed [47] by

9 $$\begin{aligned} v = {\mathrm {trace}}\left( {\mathbf {R}}{\mathbf {V}}\right) ^2 / {\mathrm {trace}}\left( {\mathbf {R}}{\mathbf {V}}{\mathbf {R}}{\mathbf {V}}\right) . \end{aligned}$$

In the present analysis, the effective DoF was approximately 30, while a normal DoF may have been as large as 1350. This example shows that modification using the effective DoF was indispensable for the NIRS–SPM analysis to avoid over-estimation in statistical computation.

*Second-level analysis* In this analysis, the statistic *t* computed by Eq. (7) was converted into a *z*-value using the effective DoF *v* and tested whether all *z*-values from all participants were statistically larger than zero for each channel (random-effects analysis).

The general form of the GLM described in Eq. (3) assumes neither a drift nor a trend effect. To fulfill this assumption, the signal is typically passed through a high-pass filter (HPF) to eliminate these effects before applying the OLS. Accurate statistical results cannot be obtained when an inadequate HPF is used. Therefore, in the following analysis, an HPF was tuned based on the Akaike information criterion (AIC). Specifically, the following three types of filters were applied based on a past NIRS study [29]: using no filter and HPFs with cutoff frequencies of .008 and .014 Hz. Table 2 shows the means of all AIC values (at the first-level analysis) and *z*-values for all 24 channels (at the second-level analysis). Each mean (and standard deviation) was computed by averaging all AICs in all channels for all participants. Similar results obtained by applying this method to oxy- and total-Hb data are also shown in gray in the same table to illustrate the trends caused by different HPFs. These findings revealed that the AIC was improved with a larger cutoff frequency. That is, the AIC obtained using a .014-Hz HPF was improved by approximately 8% compared with the other AICs calculated without the HPF. Therefore, an HPF with a cutoff frequency of .014 Hz with an effective DoF was used for the subsequent step.

**Table 2:**
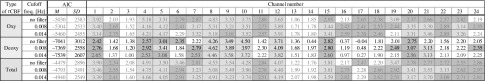
Results of statistical test using a convolution matrix method in Step 2: mean values of AIC (at a first-level analysis) and *z*-values (at a second-level analysis, df = 15)

Blue cells show $z>2.33\, (p<.01)$ and red cells show $z>3.09\, (p<.001)$. (Color table online)

### Step 3: Boxcar-function-based Gaussian convolution method

It is possible that the method used in Step 2 overestimated the actual differences because the number of channels showing significant differences in Step 2 (shown in Table 2) was roughly twice that of Step 1 (shown in Table 1). Therefore, we tested another well-used GLM approach that is also implemented in the popular SPM12 software [33]. Furthermore, we tested two modifications intended to deal with problems specific to NIRS.

In Step 3, we first assumed a GLM similar to Eq. (3) using the same boxcar function $u_k(i)$ [29]. Importantly, this differed from Step 1 in terms of the definition of an independent variable $x_k(i)$ that was convoluted by $u_k(i)$ with a Gaussian kernel function *g*(*i*) as

10 $$\begin{aligned} x_k(i)&= (g*u_k)(i)=\sum _{n}^{all} g(n) \cdot u_k(i-n) \nonumber \\g(i) &= \exp \left( -\frac{(\Delta \cdot i)^2}{2 \sigma ^2} \right) \nonumber \\\sigma &=\frac{FWHM}{2\sqrt{2ln2}}. \end{aligned}$$

The $x_k$ computed by Eq. (10) was used after being normalized with its maximum amplitude, and $x_k(i)$ for all *k* was assigned in a design matrix ${\mathbf {X}}$ as a column vector. The gray image of ${\mathbf {X}}$ is shown in Fig. 1b. To examine the response variable signal *y*(*i*), the response vector ${\mathbf {Y}}$ was composed from time-series rCBF data that were filtered using an HPF with a cutoff frequency of .014 Hz, as described in Sect. 3.2. Summarizing ${\mathbf {X}}$ and ${\mathbf {Y}}$ into a matrix equation described in Eq. (4), an estimate $\hat{{\mathbf {B}}}$ was computed using the OLS method by

11 $$\begin{aligned} \hat{{\mathbf {B}}}=\left( {\mathbf {X}}^{{\mathrm{T}}} {\mathbf {X}}\right) ^{-1} \cdot {\mathbf {X}}^{{\mathrm{T}}} \cdot {\mathbf {Y}} . \end{aligned}$$

*Modification 1: Correction of autocorrelation* Although there was no autocorrelation for the error *e* assumed in Eq. (3), this assumption was not satisfied by the actual measured data (described as Issue 1 in Introduction) [14]. For this reason, the OLS estimate is an unbiased estimator, but it is not the best linear unbiased estimator (BLUE). Hence, the statistical evaluation becomes inaccurate [30] and a type I error (“false” brain activation) is more likely to occur. Non-autocorrelation was thus recovered in Step 3 using the Cochrane–Orcutt method [48].

First, the following residual error ${\mathbf {E}}\in {\mathbf {R}} ^I$ was computed using an estimated parameter $\hat{{\mathbf {B}}}$ obtained by the OLS method without correction of non-autocorrelation.

12 $$\begin{aligned} {\mathbf {E}}= {\mathbf {Y}}- {\mathbf {X}}\hat{{\mathbf {B}}} \end{aligned}$$

Using elements $[\bar{e}(1), \bar{e}(2), \ldots , \bar{e}(I)]^{{\mathrm{T}}} := {\mathbf {E}}$ in the vector ${\mathbf {E}}$, the Durbin–Watson ratio (*DW*) was computed by

13 $$\begin{aligned} DW := \frac{\sum _{i=2}^{I} (\bar{e}(i) - (\bar{e}(i-1))^2}{\sum _{i=2}^{I} (\bar{e}(i))^2} \ \ \in [0,4]. \end{aligned}$$

Next, to examine the original *e*(*i*), a first-order autocorrelation model described by

14 $$\begin{aligned} e(i) = \rho \cdot e(i-1) + w(i) \end{aligned}$$

was assumed using a constant $\rho$ and a new signal *w* with a mean of zero and no autocorrelation. An alternative value $\hat{\rho }$ for $\rho$ was specified as

15 $$\begin{aligned} \hat{\rho } = 1 - DW/2 \end{aligned}$$

using the value of *DW* computed by Eq. (13). Finally, we performed a new OLS estimate using the following autocorrelation-corrected $x^*$ and $y^*$ from Eqs. (16) and (17). Then, the newly estimated $\hat{{\mathbf {B}}}_{2nd}$ was used in a first-level analysis of the SPM.

16 $$x^*(i)= {\left\{ \begin{array}{ll} \sqrt{1 - {\hat{\rho }}^2} \cdot x(1) &{} i = 1 \\ x(i) - \hat{\rho } \cdot x(i-1) &{} i = 2, \ldots , I \end{array}\right. }$$

17 $$y^*(i)= {\left\{ \begin{array}{ll} \sqrt{1 - {\hat{\rho }}^2} \cdot y(1) &{} \quad i = 1 \\ y(i) - \hat{\rho } \cdot y(i-1) &{} \quad i = 2, \ldots , I \end{array}\right. }$$

*Modification 2: Consideration of higher-order element* Previous research proposed that the use of the derivative component of an HRF can be effective for NIRS–GLM analysis [31]. This suggestion corresponds well with the current data because an overshoot-like waveform around the rising and falling edges appeared to be approximated by a derivative term of the canonical HRF, as shown in Fig. 3. Therefore, two new GLM models were considered: (a) a fourth-order model using a new design matrix ${\mathbf {X}}_{1d} \in {\mathbb {R}}^{I \times (4+1)}$ that included the first-derivative terms of $x_k$ described by Eq. (10); and (b) a sixth-order model using another new design matrix ${\mathbf {X}}_{2d} \in {\mathbb {R}}^{I \times (6+1)}$ that included the first- and second-derivative terms. The three models using ${\mathbf {X}}$, ${\mathbf {X}}_{1d}$, and ${\mathbf {X}}_{2d}$ were called HRF-solo (only HRF), HRF+1.d. (HRF with its first-derivative term), and HRF+2.d. (HRF with its first- and second-derivative terms), respectively.

*Total accuracy verification and optimal condition* The results obtained from the first- and second-level analyses using Modifications 1 and 2 are summarized in Table 3. This table shows the means of *DW* and AIC values relevant to all data channels from all participants. Additional results of the analyses of oxy- and total-Hb data are shown in gray to demonstrate the effects obtained by Modifications 1 and 2. Regarding AIC, the other two cases of “no correction of non-autocorrelation (No AR)” and “correction by Cochrane–Orcutt method (AR(1)), i.e., Modification 1” are shown in the same table.

**Table 3:**
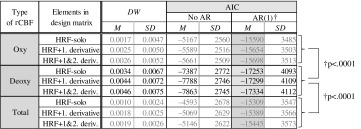
Durbin–Watson ratios and AICs when a boxcar-function-based Gaussian convolution method was applied at Step 3. (Color table online)

Examination of the values of *DW* related to Modification 1 revealed that they were relatively close to zero in all cases; hence, the assumption of non-autocorrelation of errors *e* was not satisfied. This finding supports the notion that the method used in Step 2 overestimated the statistic, because it did not involve a correction process for non-autocorrelation. The AIC indices also support potential overestimation at Step 2, since the AIC values in AR(1) were two or three times smaller than in No AR. Therefore, the current results demonstrate that correction for non-autocorrelation is indispensable for NIRS–SPM analysis, whereas several previous studies [31, 46, 47] have not taken this point into consideration. In the analysis of Modification 2, Table 3 reveals that the AIC value improved slightly when the GLM contained higher-order derivative terms. In summary, we found that a GLM model using elements of HRF+2.d. with correction for non-autocorrelation was the most appropriate for analysis of the rCBF measured during the hand-tracing tasks.

Next the appropriateness of the selection of deoxy-Hb was tested after Modifications 1 and 2 were applied. For the AIC values under AR(1) conditions shown in Table 3, differences between the deoxy-Hb and oxy-Hb groups, and between the deoxy-Hb and total-Hb groups, were investigated by Welch’s *t* test, respectively. Because each group included all AIC values computed using HRF-solo, HRF+1.d., and HRF+2.d. models, the number of samples in each group was $N= 3$ models ${\times } 24$ channels ${\times } 16$ participants $=1152$. The test revealed that the average of the AIC values computed using deoxy-Hb was significantly smaller than the average of the values computed using oxy-Hb and total Hb, as shown on the right side of the table (the deoxy-Hb vs the oxy-Hb groups: $t(1700.4)=-24.3, p<.0001$, the deoxy-Hb vs the total-Hb groups: $t(2299.9)=-4.52, p<.0001$). Therefore, it can be concluded that deoxy-Hb is better suited for analysis of rCBF since the accuracy of fit to GLM was higher when deoxy-Hb NIRS data were used. Thus, only deoxy-Hb data were used in the subsequent analyses.

We repeated the first- and second-level analyses using these modifications with the optimal conditions. The results are shown in Table 4. The results computed under conditions other than HRF+2.d., i.e., HRF-solo and HRF+1.d., are also shown in gray in the table. Contrast matrices were chosen as ${\mathbf {C}}= [\ -1\ 1\ -1\ 1\ 0\ ](K=4)$ and ${\mathbf {C}}= [\ -1\ 1\ -1\ 1\ -1\ 1\ 0\ ](K=6)$ for HRF+1.d. and HRF+2.d., respectively. Table 4 demonstrates that HRF+2.d. and HRF+1.d. were able to detect more channels showing statistical significance than HRF-solo, and there was a small difference in AIC values. Although the results in Table 4 show similar tendencies for both HRF+1.d. and HRF+2.d., we speculate that HRF+2.d. was more desirable because the AIC value for HRF+2.d. was smaller than that for the HRF+1.d. model.

**Table 4:**
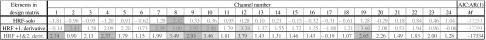
Results of statistical test using a boxcar-function-based Gaussian convolution method in Step 3: *z*-values in a second-level analysis for three kinds of design matrices, $df=15$

Blue cells show $z>2.33\, (p<.01)$, and red cells show $z>3.09\, (p<.001)$. (Color table online)

Taking the results of Sects. 3.1–3.3 together, the optimal guidelines for analyzing NIRS–SPM data for rCBF contaminated with motion artifacts can be summarized as follows:

- Type of rCBF for SPM analysis: deoxy-Hb.
- Prefilter for rCBF: an HPF with cutoff frequencies of .014 Hz.
- Method: boxcar-function-based Gaussian convolution method.
- GLM: a linear model consisting of an HRF computed using a Gaussian kernel function and its first- and second-derivative terms.
- Modifications: correction for non-autocorrelation by the Cochrane–Orcutt method and the use of effective DoF.

Note that these guidelines were obtained by considering a range of issues involved in other NIRS–SPM methods, as described in Introduction. Issue 1 (the unsatisfied assumption of normal distribution in measured data) was attenuated by optimization of statistical procedure using AIC and DW indices in Steps 1–3. Issue 2 (the non-autocorrelation problem) was resolved with Modification 1, using the Cochrane–Orcutt method in Step 3. Finally, Issue 3 (related to noise reduction) was resolved in Step 2.

## Analysis of statistical parametric maps

**Fig. 4:**
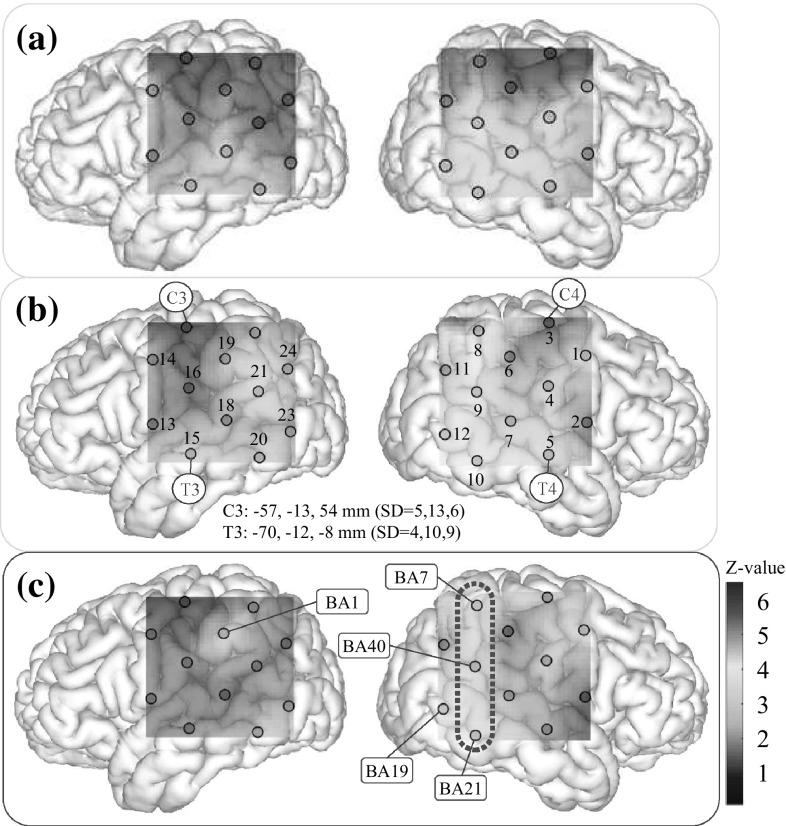
Statistical parametric maps (*z*-map images, $df=15$): **a** model-free method (Step 1); **b** convolution matrix method (Step 2); **c** a boxcar-function-based Gaussian convolution method (Step 3). (Color figure online)

The spatial distribution of brain activity associated with BS modification was investigated by focusing on the NIRS–SPM results obtained using the optimal conditions derived in Sect. 3.3. Figure 4 shows *z*-map images based on the results shown in Table 4. A *z*-map computed using the optimal conditions obtained in Step 3 is shown in Fig. 4c. This map is a visualization of the *z*-values that were described by blue numbers in Table 4. Images (a) and (b) are other *z*-maps based on results obtained in Steps 1 and 2 using the corresponding quasi-optimal conditions.^1^

The images shown in Fig. 4 are montages of a brain surface image created with the BrainBrowser Surface Viewer (v2.3.0)  [49] and the colored *z*-maps. The colored *z*-map was drawn by interpolating *z*-values with a scattered data interpolation function (in MATLAB R2015a) after deforming the positions of the NIRS channel grid with reference to C3(4) and T3(4) positions on the MNI coordinate system [50]. Circles and numbers drawn on the *z*-map image indicate the position and index of the measurement channels, respectively. Labels of Brodmann’s area numbers are provided to indicate several channels where strong significant differences were confirmed. First, Fig. 4c shows that the right hemisphere was dominant. Specifically, significant differences were confirmed in channels 1, 4, 8–10, and 12 ($z(15)>2.33, p<.01$). Channel 19, which solely indicates significant differences in the left hemisphere, is close to an area near S1(BA1), which corresponds to the tip of the finger in the cortical homunculus. This finding is in accord with the experimental circumstances, since participants used their right hand in Task 2 (while holding a long stick) more strongly than in Task 1 (which only involved one finger). Examining the positions of these significant channels revealed that the following four contiguous areas of the brain were significantly activated during Task 2: Ar1) somatosensory association cortex (BA7: $z=3.49, p<.001$); Ar2) supramarginal gyrus (BA40: $z=2.93, p<.01$); Ar3) associative visual cortex (BA19: $z=3.79, p<.0001$); and Ar4) middle temporal gyrus (BA21: $z=3.46, p<.001$). Although a similar activation pattern at Ar1–Ar4 can be seen in Fig. 4b which was obtained in Step 2, the effects in Step 2 were likely to be overestimated because the *z*-values obtained in Step 2 were larger than that in Step 3, despite the larger AIC values in Step 3. Therefore, it seems reasonable to conclude that the pattern in the *z*-map image (c) obtained by Step 3 is a feature of the BS modification related to the hand-tracing task.

Areas BA7 and BA40 related to Ar1 and Ar2 are part of the parietal association cortex. It has been found that damage to these areas causes spatial perception impairment [51]. The parietal association cortex forms the parietal lobe in combination with the S1 area, and the right parietal lobe has been closely linked to spatial skills [52, 53]. Specifically, a bimodal neuron responding to both visual and somatic senses has been reported to exist in the intraparietal sulcus (which is located near BA7) in the *right* parietal lobe [54]. In addition, the intraparietal sulcus was reported to be associated with the BS in a study of the RHI [55]. These previous findings regarding the right-hemisphere dominance of the parietal lobe are consistent with the current SPM results shown in Fig. 4c. Taken together, these results suggest that the brain areas involved in spatial perception may have been activated when BS extension was required during the use of a long stick in the current study. In addition, the inferior parietal lobule consists of BA40 and the angular gyrus (BA39),^2^ and the right inferior parietal cortex is related to own-body perception and the illusion of motion [56, 57]. Interestingly, it has been reported that out-of-body experiences [58, 59] and phantom sensations [60] can be induced by stimulating the angular gyrus, one of the areas associated with the BS.

BA19 (in Ar3) is involved in the recognition of the shape and color of objects [61]. In the present study, we speculate that this area may have become active when participants visually examined the characters traced by their fingers. This characteristic may be a feature of the BS extension because the BS is visually dominant [62]. Activation in this area, however, does not necessarily indicate general BS extension because the cognitive processing involved in recognizing Hiragana characters might have also caused neural responses in this region. Moreover, BA21 (in Ar4) is reported to be activated when subjects are engaged in contemplating distance [63]. In the current study, this brain area may have been activated during the estimation of the distance from their own body to the tip of the stick, which would be required to extend the BS spatially.

Taken together, this evidence suggests that the brain activation we observed in contiguous areas in BA7–BA40–BA21 may be related to BS extension during a hand-tracing task.

## Conclusions and future research

This study described an optimized statistical analysis procedure for NIRS–SPM analysis that involves dealing with rCBF data contaminated by motion artifacts. In addition, we identified the spatial distribution of brain activity associated with BS modification using a hand-tracing task that involved an extension of the BS. Three methodological options were evaluated in turn to determine the optimal conditions for NIRS–SPM analysis: a model-free method in Step 1, a convolution matrix method in Step 2, and a boxcar-function-based Gaussian convolution method in Step 3.

In Step 1, it was found that the actual rCBF waveform during this task could be approximated by a rounded trapezoidal waveform similar to the convolved response of a boxcar with a Gaussian function. Moreover, deoxy-Hb was found to be appropriate for the NIRS–GLM in this experiment, as indicated by the results of diagnostic screening indices concerning individual variance and AIC, which was confirmed in Step 3. In Step 2, to enhance statistical accuracy, conditions for eliminating low-frequency noise and modifying the DoF for statistical testing were investigated using the AIC. In Step 3, correction of non-autocorrelation with derivative components of HRF was applied to a GLM for SPM, by calculating the DW ratio and AIC values. Finally, credible SPM guidelines for NIRS data were obtained. Examination of the best SPM results confirmed that contiguous areas in BA7–BA40–BA21 (BA7: somatosensory association cortex; BA40: supramarginal gyrus; BA21: middle temporal gyrus) in the right hemisphere became significantly active ($p<.001$, $p<.01$, and $p<.001$, respectively) during the hand-tracing tasks, potentially representing BS modification.

Future research could incorporate the NIRS–SPM method described here to exogenously enhance the ability of BS extension using electrical stimuli.

## Footnotes

1 The quasi-optimal condition at Step 1 was “rCBF type: deoxy, time interval of integral: 10 s” because the *z*-values under this condition were largest, as shown in Table 1. The quasi-optimal condition found in Step 2 was “rCBF type: deoxy, HPF: .014 Hz” because the AIC values under this condition were smallest, as shown in Table 3.

2 The BA39 area was out of the measurable range of this NIRS setting.
